# Can urinary exosomes act as treatment response markers in prostate cancer?

**DOI:** 10.1186/1479-5876-7-4

**Published:** 2009-01-12

**Authors:** Paul J Mitchell, Joanne Welton, John Staffurth, Jacquelyn Court, Malcolm D Mason, Zsuzsanna Tabi, Aled Clayton

**Affiliations:** 1 Section of Oncology & Palliative Medicine, School of Medicine, Cardiff University, Velindre Cancer Centre, Whitchurch, Cardiff CF14 2TL, UK; 2 Cancer Services Division, Velindre NHS Trust, Velindre Cancer Centre, Whitchurch, Cardiff CF14 2TL, UK

## Abstract

**Background:**

Recently, nanometer sized vesicles (termed exosomes) have been described as a component of urine. Such vesicles may be a useful non-invasive source of markers in renal disease. Their utility as a source of markers in urological cancer remains unstudied. Our aim in this study was to investigate the feasibility and value of analysing urinary exosomes in prostate cancer patients undergoing standard therapy.

**Methods:**

Ten patients (with locally advanced PCa) provided spot urine specimens at three time points during standard therapy. Patients received 3–6 months neoadjuvant androgen deprivation therapy prior to radical radiotherapy, comprising a single phase delivering 55 Gy in 20 fractions to the prostate and 44 Gy in 20 fractions to the pelvic nodes. Patients were continued on adjuvant ADT according to clinical need. Exosomes were purified, and the phenotype compared to exosomes isolated from the prostate cancer cell line LNcaP. A control group of 10 healthy donors was included. Serum PSA was used as a surrogate treatment response marker. Exosomes present in urine were quantified, and expression of prostate markers (PSA and PSMA) and tumour-associated marker 5T4 was examined.

**Results:**

The quantity and quality of exosomes present in urine was highly variable, even though we handled all materials freshly and used methods optimized for obtaining highly pure exosomes. There was approx 2-fold decrease in urinary exosome content following 12 weeks ADT, but this was not sustained during radiotherapy. Nevertheless, PSA and PSMA were present in 20 of 24 PCa specimens, and not detected in healthy donor specimens. There was a clear treatment-related decrease in exosomal prostate markers in 1 (of 8) patient.

**Conclusion:**

Evaluating urinary-exosomes remains difficult, given the variability of exosomes in urine specimens. Nevertheless, this approach holds promise as a non-invasive source of multiple markers of malignancy that could provide clinically useful information.

## Background

Prostate cancer (PCa) remains the most prevalent male cancer in the west, with projected 186,000 new cases, and 28,000 deaths in the USA expected in 2008 (American Cancer Society, Atlanta, Georgia 2008). Whilst advances are being made in understanding the biology underlying this disease, and in many respects in its treatment, there remains a need for better tools for PCa diagnosis and monitoring.

Disease-related biomarker(s) should ideally be non-invasively available; urine-analysis fits this requirement well. Several urine-borne molecules are currently being evaluated as PCa-indicators [1-10], but recently, approaches measuring several candidate urine-markers at once may give a more complete clinical picture [11-13].

Nano-meter sized vesicles (termed exosomes) are an additional component of urine [14], which have been proposed as a possible source of multiple biomarkers of renal disease [14,15] in particular, but perhaps also of interest in urological cancer. Exosomes are a notable feature of malignancy, with elevated exosome secretion [16] and tumour-antigen enrichment of exosomes associated with cancer cells [17,18]. The physiological importance of cancer exosomes remains unclear. There are several studies suggesting they may act as an advantageous source of multiple tumour rejection antigens for activating anti-cancer immune responses [17-19]. Cancer exosomes have been proposed by some as possible therapeutic vaccines [20]. Paradoxically, however, there is also a growing number of reports demonstrating active immune-suppressive functions for cancer exosomes, assisting cancers evade immune attack [21-24]. Cancer exosomes may also contribute to angiogenic processes [25], may disseminate metastatic potential in certain settings [26] and could play roles in drug resistance [27].

From a biomarker perspective, the expression of tumour-associated antigens by exosomes naturally raises questions about the possible value of these nano-vesicles as markers of malignancy. Furthermore, exosomes may be a source of important cancer-associated antigens not available as soluble molecules within biological fluids, such as the oncofetal glycoprotein-5T4; which is over expressed by epithelial cancers but not shed from the cell surface [28]. Biological changes related to malignancy of the genitourinary tract, or to therapy, may perhaps be mirrored by changes in urinary exosomes.

In this report, we present a pilot study with the key aim of evaluating the feasibility of studying urine exosomes of PCa patients, as tools for monitoring response to treatment. Whilst we have discovered some difficulties such as variability and low quantity of urine-borne exosomes, the study provides the first encouraging evidence suggesting that further molecular analyses of urine exosomes in PCa are warranted.

## Methods

### Prostate Cancer patients and healthy donors

Ten PCa patients, participating in a local Phase II Clinical Trial, were recruited, together with 10 healthy male volunteers. The patients were confirmed positive for PCa by biopsy, and the tumour stage, Gleason score, serum-PSA and age is summarised in Table 1. Patients received 3–6 months neoadjuvant androgen deprivation therapy (ADT) prior to radical radiotherapy (RT), which consisted of a single phase delivering 55 Gy in 20 fractions to the prostate and 44 Gy in 20 fractions to the pelvic nodes. Patients were continued on adjuvant ADT according to clinical need. The trial was approved by the South East Wales Ethics Committee.

**Table 1:** Details of patients participating in this study

**Patient**	**Clinical Stage** **(all N0)**	**Gleason Score**	**Age** **(years)**	**Serum PSA** **ADT_4_** **(ng/ml)**	**Serum PSA** **ADT_12_** **(ng/ml)**	**Serum PSA** **at 6 months** **(ng/ml)**
1	T2b	7 (3+4)	66	10.5	2.10	1.2

2	T2b	7 (3+4)	62	134.0	0.20	<0.01

3	T2	8 (3+5)	70	8.3	1.40	<0.1

4†	n/d	7 (3+4)	65	83.2	83.40	†

5	T2c	7 (3+4)	69	95.2	4.10	<0.1

6	T2	8 (4+4)	70	10.8	0.10	<0.1

7	T3a	7 (3+4)	53	36.5	7.20	0.3

8	T3b	6 (3+3)	61	14.1	0.80	0

9	T2	7 (4+3)	66	21.1	0.20	<0.1

10	T2	8 (4+4)	71	28.1	1.3	n/d

† Patient died from an unrelated brain tumour prior to Radiation Treatment.

n/d not determined.

### Urine sample collection

Urine, up to 200 ml volume, collected into sterile containers (Millipore), was brought to the laboratory for processing within 30 minutes. Samples were collected mid to late morning, and these were not first-morning urine. Urine was tested for blood, proteins, glucose and Ketones and the pH was measured; (by Combur^5 ^Test^®^D, dipstick (Roche)) (summarised in Table 2). PCa-patient urine was collected at three time points: "ADT_4_" (0–4 weeks after initiation of ADT), "ADT_12_" (following three months of ADT) and "RT_20_" (after 20 fractions of Radiotherapy). At intervals during treatment (ADT_4_, ADT_12 _and at 4 weeks post Radiotherapy), serum PSA levels were measured.

**Table 2:** Details of urine specimens collected from PCa patients

**Patient**	**Time Point**	**Dip-Stick** Blood, Protein, Glucose, Ketones, pH					**Specimen Volume** **(ml)**	**Total Exosomes Recovered** **(μg)**	**Exosome Concentration** **(ng/ml)**
**1**	ADT_4_	1	1	0	0	7	90	72.9	810.0
	ADT_12_	0	1	0	0	5	180	141.9	788.3
	RT_20_	0	0	0	0	7	180	19.6	109.3
						
**2**	ADT_4_	1	1	0	0	-	170	125.5	738.2
	ADT_12_	1	2	0	0	7	180	2.61	14.5
	RT_20_	0	0	0	0	5	90	39.2	435.7
						
**3**	ADT_4_	4	2	4	0	5	180	72.9	405.3
	ADT_12_	2	1	1	0	5	180	70.9	393.9
	RT_20_	0	1	1	0	5	60	8	133.3
						
**4†**	ADT_4_	0	1	0	0	5	95	25.4	268.0
	ADT_12_	1	3	3	0	5	55	6.54	118.9
	RT_20_	-	-	-	-	-	-	-	-
						
**5**	ADT_4_	4	0	0	0	5	180	38.4	213.6
	ADT_12_	1	2	0	0	7	90	27.1	301.2
	RT_20_	1	1	0	0	6	150	5.1	34.5
						
**6**	ADT_4_	0	0	0	0	6	180	19.4	108.1
	ADT_12_	1	0	0	0	5	180	6.2	34.7
	RT_20_	1	1	0	0	5	120	9.1	76.1
						
**7**	ADT_4_	3	1	1	0	6	97	39	402.1
	ADT_12_	0	1	0	0	5	120	12.1	101.0
	RT_20_	1	1	0	0	5	45	17.7	395.1
						
**8**	ADT_4_	0	1	1	0	6	150	125.1	834.4
	ADT_12_	0	1	0	0	5	110	26	236.4
	RT_20_	1	3	0	0	7	60	34.4	574.0
						
**9**	ADT_4_	0	1	0	0	5	120	8.2	68.3
	ADT_12_	0	1	0	0	6	180	17	94.4
	RT_20_	2	3	4	0	6	60	133.1	2218.7
						
**10**	ADT_4_	0	1	0	0	5	120	19.4	162.3
	ADT_12_	0	0	0	0	7	180	11.4	63.4
	RT_20_	0	0	0	0	6	170	88.3	519.4

† Patient 4 died before RT

- Not recorded, or sample unavailable

### Exosome purification

Urine was subjected to serial centrifugation, removing cells (300 g, 10 min), removing non-cellular debris (2000 g, 15 min). The supernatant was then underlayed with a 30% sucrose/D2O cushion, and subjected to ultracentrifugation at 100,000 g for 2 h as described [17,23,29]. The cushion was collected, and exosomes washed in PBS. Exosome pellets were resuspended in 100–150 ul of PBS and frozen at -80°C. The quantity of exosomes was determined by the micro BCA protein assay (Pierce/Thermo Scientific).

### Cell culture

LNCaP and DU145 prostate cancer cell lines (from ATCC), were seeded into bioreactor flasks (from Integra), and maintained at high density culture for exosome production as described [30].

### Electrophoresis and Immuno-blotting

Cell lysates were compared to exosomes by immuno-blotting as described [31]. Primary monoclonal antibodies included mouse anti-human PSA (a gift from Dr Atilla Turkes, Cardiff and Vale NHS Trust, Cardiff), anti-TSG101, anti-LAMP-1, anti-HSP90, anti-Calnexin, anti-CD81 and anti-PSMA (from Santa Cruz Biotechnology), anti GAPDH (from BioChain Institute, Inc), anti CD9 (from R&D systems). Anti-5T4 was a gift from Dr R Harrop (Oxford BioMedica UK Ltd). Goat polyclonal anti-Tamm Horsfall Protein (THP) was from Santa Cruz, and bands were detected using anti-goat-HRP (Dako). Membranes were stripped using the Restore Plus™ western blotting stripping buffer (Pierce/Thermo Scientific), blocked overnight, and re-probed.

### Examining exosome membrane integrity

To investigate if urine damages exosome-membranes, exosomes isolated from B-cell lines, were immobilised onto anti-MHC Class-II coated dynal-beads (Dynal/Invitrogen) [32]. The exosome-bead complexes incubated overnight at 37°C in 25 mM Calcein-AM as described [31]. Calcein-loaded exosome-bead complexes were exposed to various salt-solutions or to fresh urine, at room temperature for 1 h. Fluorescence was analysed by flow cytometry (FACScan, BD), running Cell Quest software (BD). Calcein-fluorescence was compared to fluorescence of anti-Class-I (RPE) stained exosome-beads, in parallel tubes; a measure of whether exosomes remain attached to the bead surface. Results are expressed as the ratio of Calcein: Class-I fluorescence.

### Examining proteolytic damage of exosomes by urine

Exosomes purified from LNCaP cells, were treated with fresh urine in the presence or absence of protease inhibitors (including EDTA, Pepstatin-A, Leupeptin and PMSF). After 2 h or 18 h, samples were examined by western blot for expression of CD9, PSA and TSG101. As a positive control for proteolysis, exosomes were treated with trypsin (Cambrex).

## Results

### Purification of urinary exosomes

**Figure 1:**
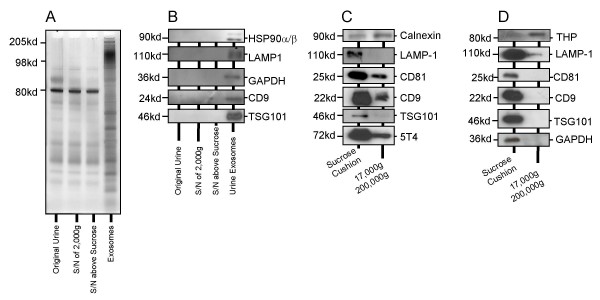
**Purification of urine-derived exosomes**. Healthy donor urine was subjected to exosome purification, and at each step, 10 μl of sample was kept for electrophoretic analysis (4–20% gradient polyacrylamide gel, silver stained) (A), demonstrating effective removal of the principal non-exosomal protein bands such as that at ~80 Kd, and significant enrichment of diverse protein species in the final exosome product (A). Parallel gels were run for immuno-blot analyses, using antibodies against typical exosome proteins as indicated (B). Comparing the sucrose cushion method, with a simpler method of Pisitkun et al, where cell culture media (C) or fresh urine (D) were subject to centrifugation at 17,000 g followed by pelletting at 200,000 g. Exosomes (from sucrose method) and the 200,000 g pellet were normalised for protein differences, and 2.5 μg/well analysed by western blot for markers as indicated.

We used a standardised method, designed for exosome-purification from cell culture supernatant, and have applied this to fresh-urine as an exosome source. With this method, exosomes are isolated based on their buoyancy characteristics [33]. Analysis of protein content of urine at multiple steps throughout purification, revealed the method was effective in eliminating principal contaminants (Fig 1a), (such as the band at 80 Kd) while significantly concentrating vesicles bearing a distinct protein repertoire, across the entire molecular weight spectrum (Fig 1a). Performing immuno-blot analyses on parallel gels revealed typical exosomal proteins were only detected in the final exosome-product (Fig 1b).

Comparing this method with the method of Pisitkun *et al *[14], using cell culture supernatants (Fig 1c) or healthy donor urine (Fig 1d) as source material, showed the sucrose method results in a pellet which is more enriched in exosomes, evident by strong band intensity for exosome markers such as CD9, TSG101 and LAMP-1. Importantly, the sucrose method resulted in good enrichment of tumour associated antigens; in this case 5T4 (Figure 1c), indicating an important advantage in analysis of exosomes over pelleted sediment [14]. Although many markers were detected in the comparator preparation, these were at a lower level. The more intense band for calnexin (a non-exosomally expressed marker), is evidence for more contaminants when using the comparator method (Fig 1c). Similarly, with urine as the source material, the sucrose-cushion method again proved advantageous (Fig 1d), showing higher levels of exosome expressed proteins, and reduced contamination with Tamm Horfsall protein (THP). The data support this approach for enriching exosomes from fresh urine specimens; and confers some advantages over previously published urine-exosome protocols.

### Changes in urine-exosome quantity during PCa therapy

**Figure 2:**
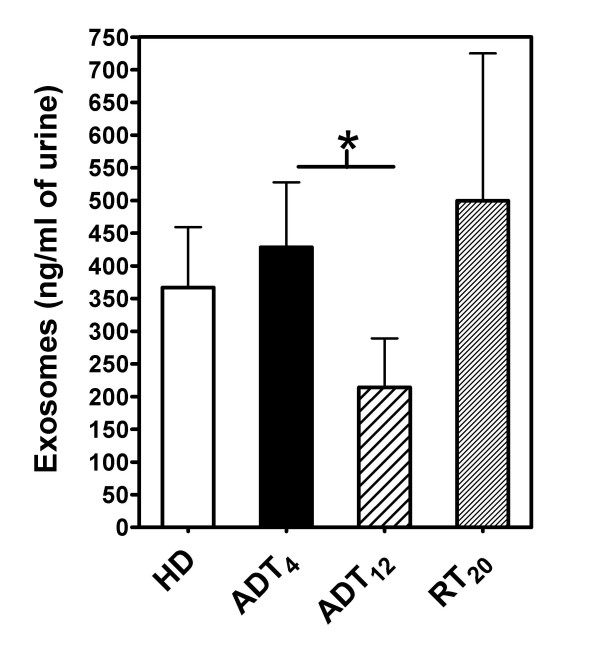
**Quantification of urine-derived exosomes, in healthy donors, and Prostate Cancer patients**. The quantity of exosomes present in each preparation was measured using the BCA protein assay. Values were corrected for urine-specimen volume, and are represented as ng Exosomes per ml of urine. Preparations from 10 healthy donors and 10 PCa patients undergoing standard therapy, at ADT_4 _(after 4 weeks ADT), ADT_12 _(after 3 months of ADT), and at RT_20 _(and after 20-fractions of radiotherapy) are compared. Bars represent mean+SE. *p < 0.5 using the Wilcoxon matched pairs test are shown.

The quantity of exosomes present in each preparation was measured, corrected for starting urine volume, and values compared across the patient (Table 2) and healthy donor (Table 3) groups are summarised in figure 2. Prostate cancer patients on average had 1.2-fold higher levels of urinary exosomes (at ADT_4_) compared to healthy men. There was broad variation in the exosome-content across both the healthy donors (366.8 ± 92.56, n = 10 mean ± SE) and patients (443.2 ± 109.7, n = 10, ADT_4_). After three months of androgen deprivation therapy (ADT_12_) there was a ~2-fold decrease in exosome levels (224.9 ± 82.7, n = 10), with 8 out of 10 patients showing a decrease in exosome quantity. In terms of radiation treatment (RT_20_, 499.6 ± 225.6, n = 9), there was no significant difference compared to ADT_4 _or to ADT_12_, as 3 out of 9 patients demonstrated a further decrease in exosome levels, whilst 6 out of 9 had increasing urinary exosome levels. There was a decrease in serum PSA levels in 9/10 patients, demonstrating that standard therapy was successful in tumour bulk reduction.

**Table 3:** Details of urine specimens collected from healthy donors

**Healthy Donor**	**Age of donor**	**Dip-Stick** Blood, Protein, Glucose, Ketones, pH					**Specimen Volume** **(ml)**	**Exosomes Recovered** **(μg)**	**Exosome Concentration** **(ng/ml)**
**1**	**29**	0	0	0	0	7	180	9.8	54.4

**2**	**37**	0	1	0	0	7	180	115.2	640.0

**3**	**37**	0	1	0	0	7	180	32.3	179.4

**4**	**63**	2	0	0	0	5	180	55.4	307.8

**5**	**61**	0	1	0	0	7	180	154.7	859.4

**6**	**50**	0	1	0	0	7	180	8.7	48.3

**7**	**49**	0	0	0	0	6	150	61.2	408.0

**8**	**55**	0	1	0	0	6	180	37.2	206.7

**9**	**56**	0	0	4	0	7	145	28.5	196.6

**10**	**57**	0	1	0	0	8	170	130.3	766.5

In conclusion it is not possible to demonstrate a correlation between locally advanced PCa with the quantity of exosomes present in urine, and there is no correlation between serum PSA and urinary-exosome levels. From the current data set, there is some suggestion however, that at ADT_12 _there is a decrease in the amount of exosomes present.

### Prostate Cancer cell lines produce typical exosomes, positive for prostate and cancer-associated antigens

**Figure 3:**
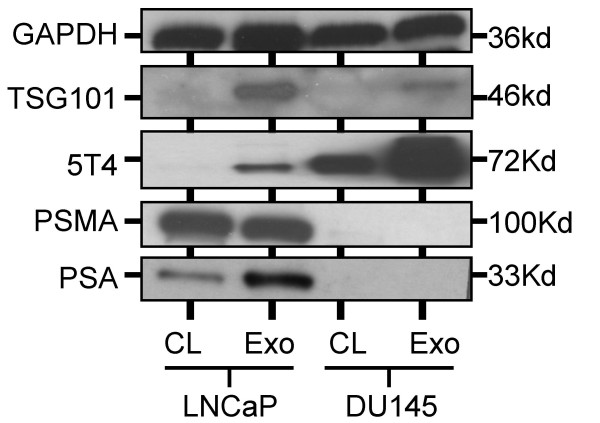
**Characterising exosomes produced by LNCaP-prostate cancer cell line**. Prostate cancer cell lines (LNCaP and DU145), as indicated, were maintained in culture as a source of positive-control prostate cancer exosomes (for subsequent analyses). Whole cell lysates (CL) or exosomes (Exo) were analysed by SDS-PAGE (5 μg/well), with a panel of antibodies as indicated.

Two prostate cancer cell lines were maintained in culture, as a source of PCa-exosomes, and the expression of typical exosome-markers (e.g. the tetraspanin CD9) and some known markers of prostate (PSA and PSMA) were examined. The LNCaP cells (whole cell lysates) were directly compared to LNCaP-exosomes by immuno-blot, revealing positive exosomal expression of PSA and PSMA. There was also clear positive exosomal expression of 5T4 by LNCaP-exosomes. Both PSA and 5T4 were particularly enriched in exosomes, compared to the parent cell (Fig 3A). The DU145 cell line, which does not express PSA or PSMA served as a control demonstrating specific staining. Staining for GAPDH showed equal loading of wells. We concluded that exosomes isolated from PCa cells express molecules typical of exosomes from other cellular sources together with prostate markers and tumour-associated antigen(s). This immuno-blot panel was considered suitable for analysis of urinary exosomes in following studies.

### The phenotype of healthy donor urinary exosomes

**Figure 4:**
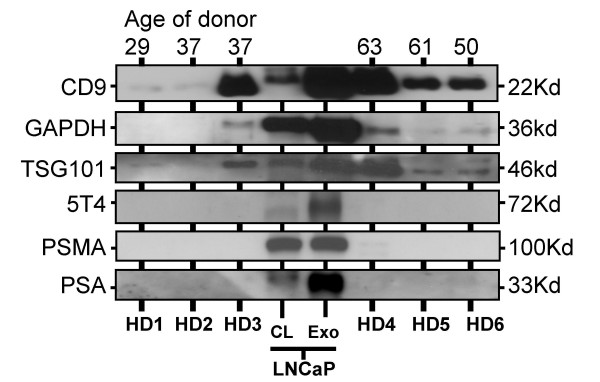
**Characterising exosomes from healthy donor urine**. Six healthy donors (detailed in Table 3), provided urine specimens and exosomes were purified. Western blots were performed with 5 μg urine-derived exosomes/well, or with 5 μg LNCaP-derived exosomes (Exo) or 5 μg LNCaP whole cell lysates (CL). Blots were probed with antibodies against PSA, TSG101, 5T4, CD9 and GAPDH, as indicated.

We performed analyses of urinary-exosomes from healthy donors (HD), and compared expression levels for these molecules to those of LNCaP-derived exosomes. Markers such as TSG101 and CD9 were detected in most HD-specimens by western blot, albeit at low levels compared to the LNCaP standard, suggesting that at least some exosomes were present in these specimens. There was considerable variability in band intensity obtained across these donors, even though analyses were all normalised for differences in protein. Prostate markers (PSA and PSMA) were not expressed in any healthy donor specimens, indicating that few if any exosomes in healthy donor urine arise from the prostate. The tumour antigen 5T4 was not found in any of the HD specimens (Figure 4).

In conclusion, examining urinary-exosomes obtained from different donors by this method is certainly feasible, and this is sufficient to reveal variation in exosome-quality across the samples. Nevertheless, in cases where exosome-quality was moderate/good (i.e. comparable to LNCaP exosomes), healthy donor urinary-exosomes could be confirmed negative for PSA, PSMA and 5T4.

### Phenotype of PCa-patient's urinary exosomes, and evaluating changes with treatment

**Figure 5:**
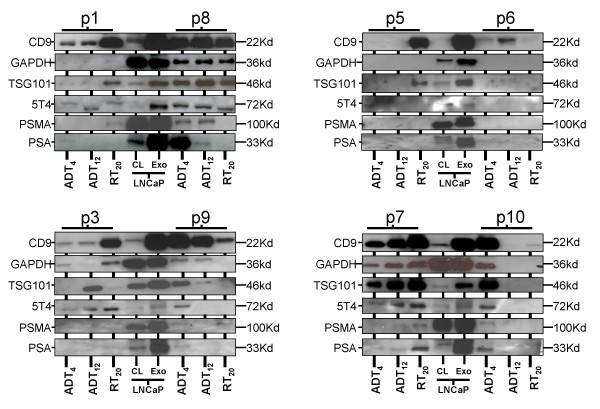
**Characterising exosomes from PCa patients**. Urinary exosomes (5 μg/well), isolated from 8 PCa patients (at ADT_4_, ADT_12 _or RT_20_), were subject to western blot analyses with a panel of antibodies as indicated. Whole cell lysates (CL) or exosomes (Exo) of LNCaP (5 μg/well) was included on each gel as positive controls.

PCa patient derived exosomes were examined in a similar manner. The data from 8 individual patients are shown (Fig 5). Overall there was variability in band intensity (with multiple markers) across the sample series, with weak staining in most occasions compared to the LNCaP-exosomes, yet there was some positivity for exosome-markers in 20 of 24 samples. There was variation across the patient cohort, and variation from within an individual's sample series (ADT_4_, ADT_12 _and RT_20_). As great attention was paid towards loading 5 μg of sample per well, we believe the results more likely reflect the variable exosomal content of the sample, rather than technical issues of sample loading. Bands for prostate-derived proteins PSA or PSMA were evident in 5 patients (p1, p7, p8, p9, p10), indicating that at least some of the exosomes present in the urine were of prostate origin. Given the variation in band intensity across the three time points in most of these samples it is not possible to demonstrate phenotypic changes in response to treatment. The exception to this is shown by patient 8, in which band intensities for exosome-markers were stable at all three time points. This patient demonstrated a strong band for PSA at ADT_4_, which diminished with treatment, becoming undetectable at RT_20_. The band for PSMA also followed this pattern to an extent, whilst the tumour-antigen 5T4 remained detectable at RT_20_, suggesting that there may be some element of residual disease present, and that exosomal 5T4 may reflect this. The data are summarised in Table 4.

**Table 4:** Summary of patient's western blot data

			**Exosome Markers**			**Cancer Marker**	**Prostate Markers **		
	
	**Patient**	**Time**	CD9	GAPDH	TSG101	5T4	PSMA	PSA	**Summary**
	
	**LNCap**	N/A	**++++**	**+++**	**+++**	**+++**	**++++**	**++++**	The Comparator "Standard" Sample
	
**Good Quality**	**p8**	ADT_4_	**+++**	**++**	**++**	**++**	**++**	**+++**	Consistent, High Quality Exosomes.
		ADT_12_	**+++**	**++**	**++**	**++**	**++**	**+**	Prostate markers diminish with treatment.
		RT_20_	**+++**	**++**	**++**	**+**	**-**	**-**	5T4 still evident at RT_20_
	
	**p7**	ADT_4_	**++**	**+**	**++**	**+**	**+**	**+**	Good quality exosomes, but inconsistent, (increasing with treatment).
		ADT_12_	**++**	**++**	**+++**	**++**	**+**	**+**	Prostate markers & 5T4 still evident at RT_20_
		RT_20_	**+++**	**+++**	**+++**	**++**	**++**	**++**	
	
**Intermediate Quality**	**p1**	ADT_4_	**+**	**-**	**-**	**+**	**-**	**+**	Inconsistent, (increasing with treatment)
		ADT_12_	**++**	**-**	**-**	**+**	**-**	**-**	Prostate markers barely detected, no clear pattern.
		RT_20_	**+++**	**+**	**++**	**+**	**+**	**-**	5T4 still evident at RT_20_
	
	**p3**	ADT_4_	**+**	**+**	**-**	**+**	**-**	**-**	Inconsistent, (increasing with treatment)
		ADT_12_	**+**	**-**	**++**	**+**	**-**	**-**	Prostate markers absent.
		RT_20_	**+++**	**++**	**-**	**++**	**-**	**-**	Strong 5T4 at RT_20_
	
**Poor**	**p9**	ADT_4_	**+++**	**+**	**+**	**+**	**+**	**+**	Inconsistent, (decreasing with treatment)
		ADT_12_	**++**	**+**	**-**	**-**	**-**	**-**	Prostate markers barely detected, no clear pattern.
		RT_20_	**+**	**-**	**-**	**-**	**+**	**-**	No 5T4 at RT_20_
	
	**p5**	ADT_4_	**-**	**-**	**-**	**-**	**-**	**-**	Poor quality at 2/3 time-points
		ADT_12_	**-**	**-**	**-**	**-**	**-**	**-**	Not Evaluable
		RT_20_	**+++**	**-**	**++**	**-**	**-**	**-**	
	
**Very Poor Quality**	**p10**	ADT_4_	**+++**	**++**	**+++**	**+**	**+**	**+**	Poor quality at 2/3 time-points
		ADT_12_	**-**	**-**	**-**	**-**	**-**	**-**	Not Evaluable
		RT_20_	**+**	**-**	**-**	**-**	**-**	**-**	
	
	**p6**	ADT_4_	**+**	**-**	**-**	**-**	**-**	**-**	Poor quality at 3/3 time-points
		ADT_12_	**++**	**-**	**-**	**-**	**-**	**-**	Not Evaluable
		RT_20_	**-**	**-**	**-**	**-**	**-**	**-**	

### Urine does not osmotically damage exosome membrane integrity

Our study highlighted variable quantity of exosomes in urine specimens. This was ~10-times lower than expected, according to others [34]. We hypothesised that variable hydration state of individuals providing urine specimens may lead to some differences in water/salt content of urine; and that this may damage exosomes present in urine. This would impact on exosome-flotation characteristics, and may explain the variability and low quantity we observed using the sucrose-cushion method.

**Figure 6:**
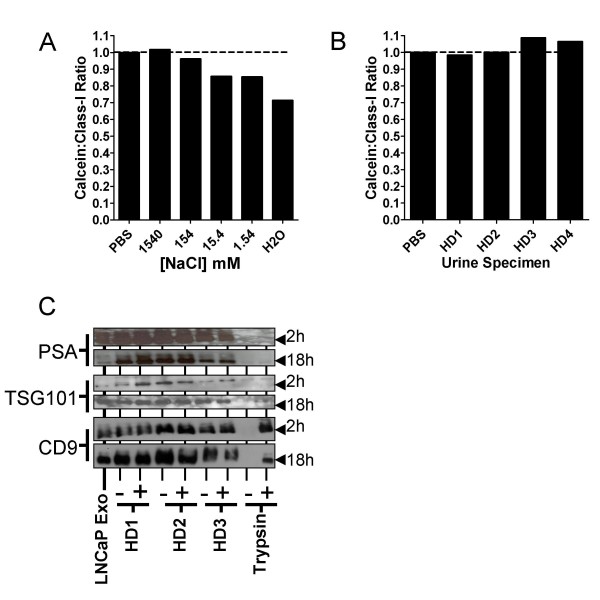
**Evaluating urine-mediated damage of exosomes**. Exosomes coupled to microbeads were labelled with a luminal fluorescent dye (Calcein-AM), prior to incubation with various concentrations of NaCl (A) or with fresh urine specimens from four healthy donors (HD1-4) (B). In parallel, identical beads were set up, in the absence of Calcein-AM dye, stained instead with anti-MHC Class-I (RPE) conjugated antibody. After 1 h at room temperature, the fluorescence signal present in the FL-1 channel (Calcein) was compared to FL-2 fluorescence (Class-I-RPE). Graphs show ratio of Calcein to Class I fluorescence. To examine proteolytic damage of exosomes (C), western blot was performed for CD9, TSG101 and PSA on LNCaP-derived exosomes; which were incubated for 2 h or 18 h with fresh urine specimens (from three healthy donors), in the presence or absence of protease inhibitors. Trypsin was used as a positive control for proteolysis.

Experiments were performed, using exosomes loaded with a fluorescent dye, to assess how various osmotic conditions might damage exosome membranes; revealing that exosomes are surprisingly resistant to high and low salt solutions (Figure 6a). Incubating exosomes in urine specimens had no impact on the integrity of the membrane (Figure 6b). We conclude that urine does not osmotically damage the exosome membrane, and this is unlikely to impact on the buoyancy characteristics of exosomes.

### Exosomes are not prone to proteolysis by urine

Proteolytic damage of exosomal constituents, by urine-proteases, may also explain low exosome levels we observed. Unlike Pisitkun *et al*, we used fresh urine specimens without protease inhibitors. To test this, we purified exosomes from LNCaP cultures, and incubated these with urine specimens in the presence/absence of protease inhibitors. Analysis of exosome markers by western blot revealed fresh urine specimens did not cause degradation of exosome-markers tested. We conclude that exosomes can largely resist endogenous proteolytic activity of urine (for at least 18 hours at 37°C) (Figure 6c).

## Discussion

We present the findings of a pilot study, investigating urinary exosomes in prostate cancer patients. We had two main aims in the study; firstly to assess the feasibility of using urine as an exosome source in the context of a clinical trial, and secondly to demonstrate changes occurring in response to standard PCa-therapy. We anticipated being able to show differences in urinary exosome quantity, between healthy individuals, and individuals with locally advanced prostate cancer, together with diminishing exosomally expressed PCa-markers in response to therapy.

Firstly, it is certainly feasible to collect spot urine specimens (up to 200 ml) from PCa patients, at multiple time points during standard treatment. The exosome purification method is laborious however, with 30 samples occupying 30-days of preparation time. This approach is not suited to larger scale trials or screening programmes, but was aimed at achieving the best quality preparations possible.

Our study highlights considerable variation in the quantity of exosomes available from spot urine specimens, and this was 10× lower than expected based on previous reports [34], where exosomes were not isolated based upon their buoyancy. Whilst some effort was invested in accounting for this discrepancy, such as evaluating the impact of urine protease activity on exosomes, or the effect of osmotic conditions on exosome membrane integrity, this discrepancy may simply be due to the presence of more non-exosomal contaminants present when using a simple pelletting approach; and that exosomes are therefore less abundant in urine than originally thought.

Comparing urinary-exosome quantity as we have done here is unlikely to provide meaningful information to the clinic, as there was no real difference between healthy men and those with locally advanced disease. We did observe a 2-fold decrease in urinary exosomes following 3-months ADT, where 8 of 10 patients showed a reduction in their urinary exosome content, and of these, 6 had reductions of >50%. This lower exosome level was not well maintained, with 5 of 9 patients showing elevated exosome levels with radiotherapy. In contrast, serum PSA levels demonstrated that all but one patient had responded well to treatment, with levels below 1.5 ng/ml at 6 months post treatment. There was no correlation between this surrogate cancer-marker, and the quantity of urinary exosomes. One may speculate that the reduction in prostate volume caused by ADT may explain the decrease in urinary-exosomes, and that radiation, a documented stimulus for exosome secretion [16], and a potent inducer of a robust local inflammatory response, may elevate exosomal urine content following radiotherapy. These aspects require further investigation.

Measuring protein quantity (present in purified exosome preparations), is clearly not sufficient to discriminate cancer cell derived exosomes, from a "high background" of non-cancer cell exosomes present in this complex mixed exosome population in urine. A future approach could involve an immuno-affinity based method, for identifying (and quantifying) the proportion of tumour marker positive exosomes present in urine. One group has previously reported an approach, based upon EpCAM expression by ovarian cancer derived exosomes, for analysing exosomes present in the circulation [35]. We and likely others are working to develop an ELISA-like approach, better suited as a screening tool for cancer-derived exosomes in urine and other body fluids. Knowledge from this study will assist us in developing this tool.

In terms of exosome-phenotype, this study has highlighted some interesting observations from some of the PCa patients' specimens. Firstly, it was not previously known that the prostate can contribute any exosomes to the total urine exosome-pool. In healthy donors there was no positive staining for the prostate markers PSA or PSMA, and the tumour marker 5T4 was also negative. In the patient cohort, PSA was evident in 8/20, and PSMA present in 9/20 specimens (where 20/24 specimens were positive for one or more exosome-markers; i.e. evaluable as exosome-positive). Staining for 5T4 showed positivity in 14/20 samples. Together, this demonstrates for the first time, expression of prostate and cancer-associated markers by urinary exosomes.

One particular patient (p8) demonstrated comparable exosomes at each of the three time points, and a clear loss of exosomal-PSA in response to therapy. Unexpectedly, 5T4 remained strongly expressed, even following 20-fractions of radiotherapy, suggesting this may be a candidate marker for assessing the presence of residual malignant cells, refractory to the effects of androgen-ablation or radiotherapy. This aspect certainly warrants follow up studies, as there is a need for markers suited to identifying the presence of treatment-resistant cells.

The future of urine-exosome analysis in prostate cancer remains uncertain. This study has demonstrated that extensive steps taken to freshly process and highly purify exosomes from urine are labour intensive, yet results in a variable product with only 17% of attempts containing exosomes of comparable quality to those obtained from cell culture. When the exosome content of source material is consistent, variation due to the preparation method used is <1% [30]. It may be possible to overcome this degree of heterogeneity in the exosome content of the source material, for example by 24 hr urine collection or by collection after prostate massage. Such modifications together with improved methods for normalisation of the sample (e.g. compare ratio of exosomes to urine creatinine for example as suggested [34]), should be adopted for future studies. Regardless of these difficulties, the urinary exosome compartment genuinely holds promise as non-invasive source of tumour-associated antigens, for PCa and likely other malignancies of the urological tract.

## Competing interests

The authors declare that they have no competing interests.

## Authors' contributions

PJM and JW equally contributed to sample preparation and analyses. JS conceived, designed and organised the study. JC provided general technical support in sample analysis. MDM assisted in study design and analysis. ZT assisted in study design, data analysis and manuscript preparation. AC drafted the manuscript and directed the overall study.
